# Letter from K. P. Moore, M. D., Macon, Ga.

**Published:** 1890-01

**Authors:** K. P. Moore

**Affiliations:** New York


*Atlanta Medical aud Surgical Journal :*

In my *last* letter from here I concluded by a promise that *this* should be in the line of my special work. For several years I have found myself gravitating into gynecology, and it is in this direction that I have devoted myself more than any other since I came to New York. If there has been any subject, the treatment of which has impressed itself upon me more than another in my contact with the various gynecologists of this city, it has been chronic endometritis; or, to be more specific, that condition of the endometrium in which the follicles or metrical glands have not only become granulated, but have degenerated into fungoid growths.

There is no question but that this pathological condition exists much more frequently than is generally supposed by the general practitioner, and a failure to recognize the true condition (and hence improperly directed treatment) is a sufficient explanation of our frequent failure to cure our patients. Perhaps it would not be just the right or true way of expressing it to say, in every instance, that the treatment was *improperly* directed, or that we *failed* to cure our patients, even when we really did not know the true pathological condition. For it is true that a cure *can* be effected by the local application to these fungous growths of the various escarotics, or, what I believe would be better, by electrolysis; but it would take a very much longer time; and what can in *this* way be accomplished in six or eight months, may, by a more radical method, be accomplished in four to six weeks.

To go back to the pathology and symptomatology of this disease: We have a patient come to us for sterility, for instance, who has dismenorrhœa and probably menorrhagia, a vaginal discharge, more or less purulent in character, perhaps a degree of vaginitis, vulvitis and concomitant pruritis; and to a greater or less extent a urethritis, causing constant and painful micturition, and perhaps a heavy, dragging sensation around the pelvic region, as if the pelvic viscera would fall out, as they often express it. Of course all of the above symptoms are not presented in every case, for there really may be but little more than the dismenorrhœa, a slight vaginal discharge, and the sterility which we may be called upon to treat. Or on the other hand, the case may not be one of sterility, but one of sub-involution of the organ after confinement or miscarriage; in which case, the uterus, whose endometrium was thus filled with this hyperplastic chronic catarrhal growth, would never involute, and might, if left alone, sooner or later be followed by disease of the appendages, or, possibly, malignancy of the uterus itself. Usually the organ is larger and heavier than normal, and its depth may be considerably increased; or, on the contrary, on account of the thickened and hyperplastic condition of the endometrium, its depth may even be shortened. The introduction of the sound almost invariably gives pain, which symptom is itself a pretty sure index of an abnormal condition; for the easy introduction of a sound into a normal uterus ought not to be painful. If, in addition to the pain, the sound should bring away bloody mucus, we may feel pretty sure of the diagnosis; but to settle the question beyond doubt, we have only to introduce one of Sims' small exploratory curettes, and by gently scraping on the walls of the uterus, we will bring away some of the enlarged fungous granulations. The cornua of the uterus seem rather to be their favorite habitat, and sometimes we may fail, as I did in one of the clinics a few days ago, to find them on the whole uterine walls, but by scraping around near either cornu, we at once remove them quite abundantly. The diagnosis having now been well made, our duty not only seems plain but, considering the strong endorsement by the leading specialists here, imperative.

At a meeting of the section on obstetrics and gynecology, of the New York Academy of Medicine, a few nights ago, this subject of curetting the uterus was well ventilated by a number of the most prominent and leading gynecologists of this city, and, with perhaps one exception, it met the entire sanction of the section, at least so far as any expression of opinion was made.

That many of these cases, even in private practice, have their etiological origin in a specific infection we do not question. Certainly a large proportion of those whom I have seen treated in the out-door poor dispensaries and free hospital wards and clinics in this city are of gonorrhœal origin. But whatever the cause the treatment is the same, when the same pathological conditions obtain. In addition to the pathological changes in the corporeal endometrium, we often find a gaping eroded cervix; especially is this apt to be the case in a sub-involution, particularly so if there is any degree of laceration of the cervix. And just in this connection I would digress a little and say, that, in my experience in the past, no operation has given me better or more satisfactory results than to repair this laceration by plastic operation, and in this opinion I am fully endorsed by the gynecologists in this city, and I would not consider any operation, or effort at treatment of laceration, complete until this had been done.

It has been a source of some amusement, as well as of profit to me, to see how widely eminent gynecologists differ upon many important questions appertaining to this branch of the practice of medicine. But however widely they differ upon many questions, it is also remarkable to note the unanimity of opinion upon the disease and its treatment, which I now have under consideration.

Briefly stated, the technique of the operation is about as follows: I should state in the beginning, that this plan of treating this condition of the uterus should be regarded as an *operation*, and should be conducted as such. While it might not be *absolutely necessary* to take all the precautions that we would in a strictly surgical procedure, yet it can do no possible harm to do so, and certainly would give ourselves the benefit of the doubt, and would place around our patient all the safeguards against possible danger. Then, I would have the vagina well douched for two or three days previous to the treatment, with some disinfecting, antiseptic solution. Perhaps one of the best, and certainly one of the cheapest and most convenient, would be the boracic acid. At the time of the treatment, it would be well to thoroughly swab out the entire vaginal canal with a fairly strong solution of bichlor. of mercury. The cervical canal should now be well dilated, so as to admit a pretty large curette, and then thoroughly curette the whole uterine cavity. The best and most permanent results seem to follow the most *thorough* curetting. I would hardly speak extravagantly to say that I had seen as much as two or three large tablespoonfuls of these fungous granulations removed at one seance. After we have thoroughly cleaned off all these fungous glands, the uterine cavity should be well swabbed out with sterilized absorbent cotton, so as to remove all the detached material, blood or other detritus, which might become a nidus of sepsis. Applications of iodine, carbolic acid, or iodized phenol, as suits the operator, can now be made; and a conical tampon of iodoform gauze, which has been first sterilized, or of absorbent cotton medicated with iron and glycerine, should now be placed into the uterus, and through the cervical canal; and several pledgets of boro-glycerine cotton packed into the vagina and around the uterus, to act both as a sort of splint or stay, and depletant; below this some dry cotton may be placed, and this, with a napkin, completes the operation. Ordinarily the procedure will have to be done under an anæsthetic, for this chronic condition has superinduced a high state of hyperæsthesia and sensitiveness, which will make the operation one accompanied with a good bit of pain. Still, the fortitude and tolerance of pain exhibited by most women is wonderful, and many of them will go through the operation with but little complaint.

The patient should now be put to bed and kept there for several days. Munde, and others, at once apply an ice bag to the abdomen, which is kept on for twenty-four to forty-eight hours. This they recommend as a matter of precaution against possible inflammatory trouble. Should there be sufficient oozing to saturate the dressing, it should all be removed within twenty-four hours, or even sooner; and if hemorrhage to any extent is found to exist, styptics to the intra-uterine cavity can be applied.

The subsequent management of the case would be such as would suggest itself to every intelligent physician. It may require two or three intra-uterine medications at intervals of a week; large, hot vaginal douches to be taken twice per day; vaginal packings of boro-glycerine tampons, etc., etc.

Usually this operation gives new life and vigor to the whole organ. Healthy granulations at once spring up, sub-involution begins, and in a few weeks the uterus is restored to its normal condition. Of course, free drainage should be kept up, and, if necessary, an occasional dilatation made.

The question might well be raised as to whether it is best to use the sharp or dull curette. This is a question which every one must answer for himself, and, indeed, each case, in proportion to the intensity or severity of the pathological condition, must indicate as to whether it is best to use the dull or sharp instrument. The preponderance of authority here seems decidedly in favor of the sharp curette. Of course, no very appreciable or unnecessary amount of force should be used; we merely aim to scrape off the hyperplastic granulations, and it does not require a great deal of force; and yet it would be better, if an error should be made at all, that it should be made in favor of too much rather than too little scraping. It would, perhaps, do less harm to do slight injury to the sound mucous membrane than to allow any of these fungous growths to remain.

It is hardly necessary to state that the patient should be put in Sims' position, and a Sims' speculum used, and a tenaculum hooked into the anterior lip of the cervix, so as to bring the organ within easy reach, and to hold it steady.

It would not be out of place here to mention some of the contra-indications for this treatment. It would hardly be a prudent operation in a case of subacute pelvic peritonitis, even though it should be very circumscribed; nor would it hardly be safe in a case of constantly recurring acute attacks of pelvic peritonitis, with more or less adhesions binding down the uterus, even if there existed, at the time, no inflammatory condition. For we would not want to take the risk of lighting up what might be a smouldering predisposition to an inflammation. Nothing has been said of constitutional treatment. This hardly comes within the scope of this paper; for it has been my purpose to emphasize the curette as a remedial agent in treating chronic endometritis, more than to go into the general management of the case. Certainly no intelligent physician will overlook the proper care of his patient's general condition, and the judicious administration of constitutional and hygienic treatment is as important as the local treatment. These go hand in hand, and neither will afford the best results without the help of the other. It goes without saying that good air, nutritious food, tonics, etc., should receive due attention.

*New York, Dec. 1st, 1889.* K. P. Moore.

